# Family involvement in forensic psychiatric care: a professionals’ perspective

**DOI:** 10.1192/j.eurpsy.2022.890

**Published:** 2022-09-01

**Authors:** S. Rowaert, S. Vandevelde, G. Lemmens

**Affiliations:** 1 Ghent University, Department Of Special Needs Education, Ghent, Belgium; 2 Ghent University, Department Of Head And Skin, Ghent, Belgium

**Keywords:** Family involvement, Professionals’ perspectives, forensic psychiatry, Qualitative research

## Abstract

**Introduction:**

Research shows that family members of forensic patients often have the feeling not to be sufficiently involved in the treatment and care trajectories of their relative. Also professionals indicate to encounter several barriers to involve family members, including lack of time and skills, organizational barriers and meddling family members.

**Objectives:**

This study aimed to map professionals’ reflections on family involvement in forensic psychiatric care. The research questions related to how professionals experience family involvement in forensic care and what needs to change in the future? A specific focus is placed on changes in their perspective over time.

**Methods:**

Findings of focus groups administered in 2015 with professionals working in forensic psychiatric care were supplemented with interview data collected in 2021.

**Results:**

The results show that there are several differences in how professionals experience and look at family involvement in forensic psychiatric care. Where in 2015 the question often was raised about what can be done as a professional for family members, professionals now more refer to the added value of family involvement for both the forensic patient and his/her care trajectory.

**Conclusions:**

The past six years, there seemed to be an evolution in how professionals experience the involvement of family members in forensic psychiatric care, that is increasingly perceived as valuable. Yet, the professionals indicated that challenges remain regarding professional confidentiality and shared decision making.

**Disclosure:**

No significant relationships.

